# Searching for New Human Behavior Model in Explaining Energy Transition: Exploring the Impact of Value and Perception Factors on Inconsistency of Attitude toward Policy Support and Intention to Pay for Energy Transition

**DOI:** 10.3390/ijerph191811352

**Published:** 2022-09-09

**Authors:** Yoonjung Oh, Seoyong Kim, Sohee Kim

**Affiliations:** 1 Research Center for Energy Transformation Policy, Social Science Research Institute, Ajou University, Suwon 16499, Korea; 2 Department of Public Administration, Ajou University, Suwon 16499, Korea

**Keywords:** energy transition, attitude toward policy support, intention to pay, attitude-intention consistency, value

## Abstract

The purpose of this study is to compare and analyze the factors influencing the public’s attitude toward policy support and intention to pay for energy transition from nuclear to renewable energy. We focus on inconsistency issues between attitude and intention. To this end, we set the attitude toward policy support and behavioral intention to pay as dependent variables, and value factors (i.e., ideology, political support for the current Moon Jae-in government, environmentalism, and science-technology optimism) and perception factors (i.e., perceived risk, benefit, knowledge, and trust) as the independent variables. Based on a survey, the analysis showed that at the variable level, the perceived benefits and trust in renewable energy and perceived risks and benefits in nuclear energy influenced the attitude toward policy support and the intention to pay for energy transition. Second, when evaluating the explanatory power of independent variables, the attitude toward the energy transition was affected in the following order: (1) perceived benefit in nuclear power (β = 0.259) > (2) perceived benefit in renewable energy (β = −0.219) > (3) perceived risk in nuclear energy (β = 0.202) > (4) Moon Jae-in government support (β = 0.146). On the other hand, behavioral intention to pay for energy transition was influenced in the following order: (1) trust in renewable energy (β = 0.252) > (2) Moon Jae-in government support (β = 0.154) > (3) perceived risk in nuclear energy (β = 0.139) > (4) perceived benefit in renewable energy (β = 0.099). Third, variables such as environmentalism, perceived benefit/risk/trust in renewable energy, and perceived benefit/risk in nuclear energy affected inconsistency between attitude toward policy support and intention to pay for energy transition.

## 1. Introduction

The purpose of this study is to compare and analyze the factors influencing the attitude of the public toward policy support and intention to pay for the transition from nuclear to renewable energy. Since the Fukushima nuclear accident in Japan in 2011, many countries have tried to change the energy system from nuclear power to renewable energy. After the Paris Agreement in 2016, 121 countries have signed a climate alliance with a carbon-neutral goal, and energy transition has since become a hot topic worldwide. Accordingly, in Korea, after the Moon Jae-in administration took office in 2017, the Korean government has pursued energy transition policies based on “de-nuclear power” and “expansion of renewable energy”. Recently, owing to the controversies over 100% renewable energy or “RE100” and “de-nuclear power plant” in the presidential election and the experience of soaring electricity and LNG prices, the public interest in energy transition has increased even more.

Social survey has shown a positive awareness from the public toward the energy transition to renewable energy. According to the 2017 “People’s Attitude Survey on Nuclear and Energy Policies” conducted by the Hyundai Economic Research Institute [1], 84.6% of the respondents actively support the expansion of renewable energy generation and support the environmental energy policy. Further, in 2019, the Korea Energy Information and Cultural Foundation found in a social survey [2] that 84.2% of the respondents agree on the need for an “energy transition policy” to reduce nuclear power plants and increase the proportion of renewable energy. This survey confirmed that the need for a transition to renewable energy policy is consistently high. 

However, the energy transition to renewable energy may in the short term cause an increase in energy prices, increasing the economic burden. For example, energy prices may have risen by decreasing the share of electric generation by nuclear power, evidenced by the results from a survey conducted by the Korea Energy Information and Cultural Foundation [2], where the respondents highlighted that the energy price increase (33.6%) was the most negative effect from the energy transition policy. On the other hand, a survey by the Hyundai Economic Research Institute [1] found that people had the intention to pay in increase in electricity rates for transition to environmentally friendly energy. However, because not everyone agrees to pay, conflicting opinions will arise over the cost burden of energy transition; public had positive or negative attitudes toward various policy proposals to increase the supply and expansion of renewable energy and acceptability of nuclear energy [3,4,5,6,7].

Despite the formation of a social consensus on energy transition, the expected costs create reluctance to an energy transition that brings out the differences between attitudinal support and the cost burden. Therefore, the attitude of supporting the policy does not always incur costs. If there is any inconsistency between support for energy transition and the cost burden, the process of transition will not be easy. Since such an inconsistency is important during energy transition, empirical studies are required.

This study aims to compare and analyze the influencing factors between the attitude toward policy support and intention to pay for the energy transition, focusing on the difference of the determinant structure between policy support for energy transition that does not bear costs and intention to pay for costs. When setting the predictor for two predicted variables, we considered that studies in policy acceptance lacked an integrated approach. Therefore, this study focuses both value and perception factors, which have been separately considered previously [8,9,10,11]. 

Section 2 describes briefly the energy transition policy in Korea. Section 3 carefully examines the practical issues of energy transition policies, theoretical backgrounds, and hypotheses. Section 4 describes the survey design method and questionnaire used in this study. Section 5 presents the multiple regression and logistic analysis results to show the determination structure of policy support and intention to pay. In particular, the inconsistency between policy support and intention to pay costs for energy transition through logistic regression is analyzed. Finally, Section 6 describes the results of the study and attempts to derive practical implications.

## 2. Short Description of Energy Transition Policy in Korea

Energy transition refers to the shift to new eco-friendly renewable energy as a new energy source to replace existing fossil fuels (e.g., oil, natural gas, coal) and nuclear power. To respond to climate change, the change from fossil fuel energy to renewable energy as the mission strategy must be accepted by the public sector as well as private companies. As of May 2021, 300 global companies participate in RE100, which demands companies replace their present energy sources with 100% eco-friendly renewable energy [12]. According to the “Renewable Capacity Statistics 2021” of IRENA [13], renewable energy facilities are continuously growing with the addition of a total of 261 GW in 2020. As such, the policy of energy transition from old fossil-based energy to renewable energy began in advanced countries such as Germany, Japan, the United Kingdom, and France. Energy policies in developed countries have focused on the increase in the proportion of renewable energy and natural gas and the priority on highly efficient energy consumption. Energy transition means not only converting fossil fuels into more environmentally friendly energy, but also converting geothermal heat, precipitation, sunlight, and water into new energy forms such as hydrogen energy and fuel cells. This energy transition is being used worldwide to reduce greenhouse gas and air pollution [3].

Since the renewable energy policy was first introduced in 1997, Korea has begun to supply and spread renewable energy to reduce its greenhouse gas and air pollution in accordance with the global transition trend to eco-friendly energy [3]. In 2010, the Framework Act on Low Carbon Green Growth was enacted to organically link and integrate efficient and systematic green growth policies with a focus on climate change, energy, and sustainable development. On this basis, the Moon Jae-in government revised the Article 39 of the Framework Act on Low Carbon, Green Growth in relation to energy policy [14]. The government formulated and implemented energy policies and plans to promote low-carbon green growth, with the direction of transition from de-nuclearization to renewable energy expansion. Finally, the Moon Jae-in government established an energy transition roadmap to lay the foundation for a transition to clean and safe energy.

The Energy Basic Plan is a comprehensive plan for energy policy in Korea. The 2nd Energy Basic Plan (2002–2011) aims to promote new consumption patterns, expansion of distributed and participatory energy systems, harmony between the environment and safety, and energy transition/energy mix, and to strengthen the global competitiveness of the energy industry. In detail, this includes (1) converting a supply-oriented energy system into a high-efficiency and low-consumption structure through innovation in a consumption structure, (2) solving fine dust problems and enabling greenhouse gas reduction obligations, (3) expanding the participation in large-scale centralized energy facilities, and (4) fostering new industries and services [15]. In June 2019, Korea promoted the 3rd Energy Basic Plan (2019–2040), which includes a blueprint for national energy transition by 2040. According to the Ministry of Trade, Industry and Energy [15], this plan aims to achieve a transition to clean and safe energy by reflecting the needs of the times while maintaining consistency with the basic directions of the first and second plans. This plan, designed with the collaboration of experts through working groups and the public, public discussions, and meetings, has set a direction to reflect the “transition to clean and safe energy”. While the 2nd Basic Energy Plan emphasized the efficient use and stable supply of fossil fuels, the 3rd Basic Energy Plan proposed expanding the proportion of renewable energy to 30–35% by 2040 through gradual and drastic reduction in nuclear power and coal power. This implies that the main energy source will be conversion from the existing nuclear power plant and coal center to renewable energy.

However, reducing the use of existing coal and nuclear power plants and increasing the proportion of renewable energy generation will change not only the relative price between energy sources, but also each energy intensity, power generation technology, and scale [16]. Since the power generation cost by nuclear power and coal power generation was low, the unit price would rise with energy system transitions to LNG and renewable energy. In addition, if electricity demand itself is reduced, energy demand improvement will consequently lead to a desired greenhouse gas reduction. However, practical difficulties in the short term may exist due to undervalued electricity rates, while an increase in power rates is feasible in the long-term depending on the renewable energy. 

In this regard, it is necessary to evaluate whether the people have the behavioral intention to pay the cost of energy transition. The Hyundai Economic Research Institute survey [1] reported that the public intends to pay KRW 15,013 per month as an additional cost for energy transition, an increase of 9.7% from the previous survey (KRW 13,680 per month). Further, according to a survey about energy transition by the Korea Energy Information and Culture Foundation [2], there was a positive attitude toward public safety (31.3%) and eco-friendly system construction (23.7%), but a negative one toward energy price (33.6%) and unstable energy supply provision (7.2%). The negative attitudes will decrease the intention to pay for the energy transition, finally reducing the acceptance for the energy transition policy. 

## 3. Theoretical Background, Research Model, and Hypotheses

### 3.1. Attitude and Intention to Action

**Figure 1:**
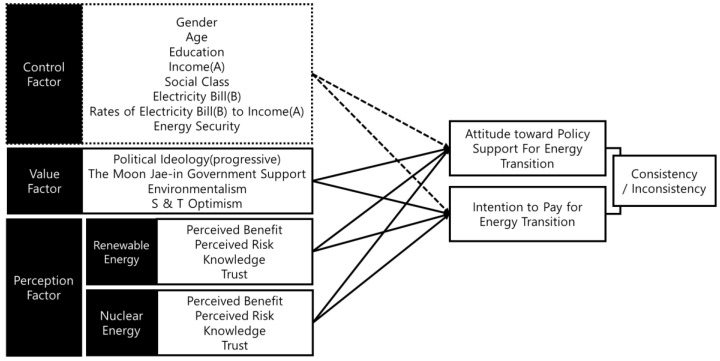
Research model.

This study aims to compare and analyze the effects of two independent factors, i.e., value, perception on the attitude toward policy support, and intention to pay for energy transition. In this study, energy transition is defined as change from nuclear energy to renewable energy. We focused not only on the transition from fossil energy but on one from nuclear energy to renewable energy given that there have been more conflicts over the latter than the former. The analysis model of this study is shown in Figure 1.

Within the independent factors, values and perception factors have very different properties. The former refers to people’s fundamental orientations that do not change easily, while the latter refers to superficial beliefs or feelings that can be changed immediately according to the external stimuli. 

In this study, the value factor consists of five variables, such as political ideology (progressive), the support for the current Moon Jae-in government, environmentalism, and science and technology optimism (hereafter referred to as S&T optimism). Values and norms are variables that influence behavior intentions and behaviors. Ajzen and Fishbein [17] demonstrated that not only attitudes toward behaviors but also their normative beliefs about behaviors were highly associated with intentions to perform these behaviors. Moreover, Ihemezie et al. [18] showed that human values influence attitudes and behaviors toward forest conservation. Further, various studies have empirically tested that political ideology [19], environmentalism [19], and S&T optimism [20] influenced the attitude toward energy, e.g., renewable and nuclear power energy [19,20,21].

The perception factor is composed of perceived benefits, perceived risks, knowledge, and trust. The perception factor depends on theoretical assumptions from the psychometric paradigm, i.e., the risk perception paradigm [22]. This paradigm was constructed by Paul Slovic, Baruch Fischhoff, and Sarah Lichtenstein and argued that risk is a subjective multidimensional phenomenon [23]. The traditional approach to measure risk was through systematic calculations for the objective frequency or severity of risk, but these methods have the limitations of not explaining the subjective perception of risk of an individual [24]. Even if the probability of a risk occurring is low, individuals can subjectively calculate this probability as high. Although the number of car accidents is higher than that of nuclear power plant accidents, people regard the latter as the highest risk. Accordingly, the risk perception paradigm approached risk in two ways based on expressed preferences. First, the reality of risk is assumed as a subjective concept, not an objective reality. According to Slovic [22], it is difficult for the public to fully understand the quantitative information of the risk and rather rely on the qualitative characteristics of the accident. Since risk is a concept perceived subjectively, even if it is the same concept, individuals can perceive it differently [25]. Second, this paradigm focuses on public opinion, i.e., social and psychological aspects, as well as on traditional, technical, and physical aspects in relation to calculating the risk [26]. According to Chaffee and McLeod [27], the perception or evaluation of an object is influenced by the perception or evaluation from other social members. Therefore, the perception of risk of an individual depends not on technical and physical but on social and psychological aspects. Risk perception is socially constructed by sharing the meaning of risk during social interactions among people. Therefore, when determining an individual’s attitude toward energy, it is often found to be decided by the perceptions of other members of society rather than on the beliefs of the individuals themselves [28].

For the dependent variables, this study focused on attitude and intention to pay because their attributes and determinants are different. Policy support is closely connected with attitudes before actions, whereas intention to pay is highly associated with a behavior. The relationship between attitude and intention has been widely studied. As a seminal research work, Fishbein [29] showed that attitude was correlated with intention to action. Ajzen and Fishbein [30] demonstrated that not only attitudes toward acts but also normative beliefs with respect to these behaviors influenced behavioral intentions for single acts, as well as for acts in dichotomous and multiple-choice situations as a function. Similarly, Ajzen and Fishbein [31] showed that when attitude toward a risky act and normative belief predict behavioral intentions, the former act carried more weight than the latter in determining intentions. According to Ajzen and Fishbein [32], these intentions are in turn a function of two components: (a) the attitude toward the act in question and (b) the perceived normative expectations from reference groups, multiplied by the motivation of a person to comply with the expectations. Variables other than these two components affect behavioral intentions and overt behaviors indirectly by influencing one or both components. Finally, Ajzen and Madden [33] experimentally demonstrated that the theory of planned behavior showed more accurate prediction of intentions and goal attainment than did the theory of reasoned action. Additionally, Ajzen [34] explained that intention to perform different kinds of behaviors can be predicted with high accuracy from attitudes toward the behavior, subjective norms, and perceived behavioral control. These intentions can also account for considerable variance in actual behavior.

Based on a meta-review covering relationships between attitude, intention and behavior, Albarracín et al. [35] reported that (a) intentions were related to condom use behavior (weighted mean r = 0.45), (b) attitudes (r = 0.58) and subjective norms (r = 0.39) predicted intentions, and (c) behavioral beliefs were associated with attitudes (r = 0.56) and normative beliefs were associated with norms (r = 0.46). The attitude does not necessarily guarantee the intention, since the latter involves costs, which makes the relationship between attitude and intention inconsistent. Fishbein and Ajzen [36] demonstrated that there was weak correlation (0.17) between attitude toward religion and each of the 100 intentions to perform various religious behaviors. Moreover, the inconsistency might have relationships with the differences in influencing factors. Moreover, Bentler and Speckart [37] demonstrated the models that intentions can be directly affected by factors other than attitudes, in contradiction to the argument of Fishbein and Ajzen. These results raise the need for comparative analysis of the determinants of attitude and intention.

Attitude toward policy support refers to a positive attitude expressed through personal evaluation of a policy [38]. On the other hand, the intention to pay, which refers to the optimal payment behavior of a consumer for a good, and an evaluation method that quantifies the net utility and value inherent in consumer consciousness, are somewhat different from the actual payment by the consumer [39]. Attitude toward policy support is closely related with more ideological issues that do not incur costs but can lead to actions, whereas behavioral intention to pay is associated with economic issues that lead to actions while incurring costs. Both concepts contain the intention to elicit action. However, there is a difference in whether or not they can tolerate the introduction of costs. 

Moreover, various studies have evaluated the determinants of intention rather than those of attitude. Based on a meta-review, Conner and Armitage [40] showed that salient belief measures, past behavior/habit, perceived behavioral control (PBC), self-efficacy, moral norms, self-identity, and affective beliefs explained intention. On the other hand, one’s attitude of a person toward an object is a function of his/her beliefs about the attributes of the object and the evaluation of those attributes [41]. However, few studies have compared the determinants of attitude and intention, focusing on the consistencies and inconsistencies between them. Therefore, we hypothesize that (1) there will be consistency and inconsistency between attitude and intention for the energy transition, and (2) the determinant structure for attitude and intention to pay will be different for the energy transition.

**Hypothesis** **1** **(H1).** *There will be not only consistency but also inconsistency between attitude toward energy transition and intention to pay for it.*

**Hypothesis** **2** **(H2).** *The determinant structure of attitude toward policy support and intention to pay for energy transition will be different.*

### 3.2. Value Factor

#### 3.2.1. Political Ideology

Political ideology is defined as a belief system that represents the direction of functionally connected values and structured attitude [42]. Political ideology provides shared values and beliefs by people looking at and responding to one’s environment [43,44]. Besley and Oh [45] showed that being liberal is generally related with less support for nuclear energy in the USA. Nuclear power is favored by the supporters of conservative political parties in Korea [46], whereas progressive parties have a high intention to pay taxes for producing energy through renewable energy resources [47]. Similarly, progressivism induces a preference for new and renewable energy [48]. According to a study by Lee [49], conservatives were interested in the need for nuclear power for economic growth and material prosperity, whereas liberals were interested in the development of eco-friendly alternative energy. 

Populus [19] studied the relationship between renewable energy and ideology, where conservative individuals showed high support for new nuclear power plants and low support for new renewable energy. From these results, it can be inferred that the more progressive people are, the more positive toward the energy transition to renewable energy. Hence, the following hypothesis can be made:

**Hypothesis** **3** **(H3).** *The more progressive the political ideology is, the more positive the attitude and intention toward the energy transition from nuclear power to renewable energy.*

#### 3.2.2. Government Support 

Not only political parties but also the president influence the way citizens support climate and energy issues [46,50]. In countries adopting a presidential system, the president is a source of public support for policies, not only on a symbolic level, but also at a practical policy level. The policy promises made before and after a presidential election power are like a product for voters in the political market. In Korea, the energy transition became a hot topic in the 2017 presidential election. Candidate Moon Jae-in was in favor of the energy transition, pledging to stop the construction of new nuclear power plants and making a transition to new and renewable energy. After becoming president, the construction of new nuclear power plants was stopped and the implementation of the plan for energy transition to renewable energy was announced. In the USA, since the Republicans favored fossil fuels, they choose not to provide any specific target for developing renewable energy, whereas the Democrats have moderate to ambitious goals for developing solar and other renewable energy [51]. President Biden has implemented a bold policy action to increase domestic clean energy by making clean energy investments, tax cuts, and federal procurement for it. Therefore, we can hypothesize the following:

**Hypothesis** **4** **(H4).** *The more support people give to the government of President Moon Jae-in, the more positive attitude and payment intention will exist for the energy transition from nuclear power to renewable energy.*

#### 3.2.3. Environmentalism

Environmentalists are concerned with nature and the entire ecosystem rather than humans [52]. Environmentalism stresses that humans have a duty to respect plants, animals, and nature. According to a study by Corner et al. [53], eco-friendly values have a negative effect on the support for nuclear power. Wang and Kim [54] demonstrated that at the contextual level, not only the share of the energy supply by nuclear power but also environmentalism influenced the acceptance of nuclear energy. Besley and Oh [45] showed that the accident at Fukushima had an impact on the attitude toward energy; that is, the environmental views and political ideology over time moderated the attention to energy issues. On the other hand, Mozumder et al. [20] showed that environmental consciousness plays a role in inducing the intention to pay for renewable energy. Based on a survey of residents in the U.S. Southwest, Carlisle et al. [55] reported that environmentalism is significantly associated with the support for the development of solar energy. Based on these studies, we hypothesized the following:

**Hypothesis** **5** **(H5).** *The higher the environmentalism people have, the more positive attitude and payment intention they will have for the energy transition from nuclear power to renewable energy.*

#### 3.2.4. S&T Optimism

S&T optimism is the positive evaluative attitude toward the function and role of S&T. Science and technology have solved many social problems; in relation to the environment, science and technology are regarded as a means of solving environmental problems [56]. These views can be applied to the energy sector, as high confidence in S&T delivers optimistic views that it will be able to overcome energy problems, including shortage, pollution, and climate change caused by carbon energy [57]. Kim [48] argued that S&T optimism will positively affect policy support and payment behavior for renewable energy. The Fukushima nuclear accident has raised public doubts regarding the role of S&T in preventing accidents. Today, advances in S&T are facilitating the transition to renewable energy. Based on these discussions, the following hypothesis is proposed:

**Hypothesis** **6** **(H6).** *The higher S&T optimism people have, the more positive attitude and payment intention they will have for the energy transition from nuclear power to renewable energy.*

### 3.3. Perception Paradigm

#### 3.3.1. Perceived Benefit

Perceived benefit showed the greatest influence on the acceptability of specific energy, thus emerging as a key predictive factor of the social acceptability of controversial electricity generation [58]. The higher the perception of the benefits from either nuclear or renewable energy sources, the higher the acceptance of energy acceptability. In the case of nuclear energy, Visschers and Siegrist [59] demonstrated that perceived benefits have a positive effect on acceptance. Besley and Oh [45] showed that when nuclear energy is perceived as less beneficial and riskier, federal and private sector decision makers are seen as less procedurally fair and the public will be less accepting of new nuclear energy plants, even more so after Fukushima. The magnitude of the perceived benefits can vary among countries, and energy security can be used as an important criterion for judging the benefits, including energy dependence. Korea is highly dependent on nuclear power as one-third of the electricity is supplied through nuclear power generation [60]. 

Considering renewable energy, Carlisle et al. [55] demonstrated that providing developers with considerable incentives is significantly associated with the development of solar energy. Based on a meta-analysis of acceptance of solar energy generation facilities, Schulte et al. [61] demonstrated that adoption intention was influenced by both benefits and perceived behavioral control (R^2^ = 0.280); such benefits in turn could be predicted by environmental concern, novelty seeking, and subjective norm (R^2^ = 0.641). Further studies have focused on perceived costs as opposed to perceived benefits. Park and Ohm [62] demonstrated that the perceived cost of renewable energy technologies was the largest variable in explaining the variance in the intention to use the technologies. 

Acceptance of nuclear and renewable energy depends not only on the perceived benefit of targeted energies but also on the perceived benefit of the other energies being compared. That is, an increase in the perceived benefits of renewable energy increases its acceptance, which is facilitated when the perceived benefits of nuclear energy are low. The same logic applies to energy transition. Hence, the following hypothesis is proposed:

**Hypothesis** **7** **(H7).** *The higher the benefit people perceive for renewable energy and the lower benefit they perceive for nuclear energy, the more positive attitude toward policy support and payment intention they will have for the energy transition from nuclear power to renewable energy.*

#### 3.3.2. Perceived Risk

The perceived risk felt by the public in relation to specific energy plays a role in lowering the acceptability of such energy and, consequently, leads to a passive attitude toward energy transition. Regan and Fazio [63] demonstrated that those who had a direct experience with a crisis show greater consistency between their attitudes and behavioral attempts. Since the Fukushima nuclear accident in Japan in 2011, the perception of nuclear power has negatively changed, and the acceptance of nuclear power has declined [56,64,65]. Besley and Oh [45] showed that after Fukushima, since respondents had seen energy as less beneficial and riskier, they revealed less acceptance of new nuclear energy plants. 

In the case of renewable energy, the perceived risk has a negative effect on its acceptability [62]. After comparing the determinants of predicting acceptance toward energy sources such as fossil fuel, nuclear energy, and hydroelectricity, Bronfman et al. [58] showed that the perceived risk had a direct effect on judgments regarding social acceptability. Park et al. [62] showed that both perceived risk and benefit had direct effects on the acceptance of renewable energy technologies.

**Hypothesis** **8** **(H8).** *The higher the risk people perceived for renewable energy, and lower risk they perceive for nuclear energy, the more positive attitude toward policy support and payment intention they will have for the energy transition from nuclear power to renewable energy.*

#### 3.3.3. Knowledge

Knowledge is a major resource that can affect risk judgment. Knowledge variables are useful in explaining acceptance and can functionally contribute to better acceptance by increasing it [66]. Generally, the higher the knowledge of the risk, the lower the individual-level risk perception [67]. Under those situations, those lower perceived risks positively influence the acceptance of the risky object. Greenber [68] confirmed that high knowledge of nuclear power was associated with high support for nuclear energy. Similarly, Ko et al. [8] showed that the objective level of knowledge had a positive effect on the economic perception and acceptance of nuclear power. On the other hand, Mok [10] confirmed that the more people were aware of the risk of nuclear power, the more negative effect there is on policy acceptance. 

In the case of renewable energy, according to Park and Ohm [62], knowledge indirectly influences the acceptance of renewable energy by way of perceived risks and benefits. After comparing Germany and New Zealand, Langer and Wooliscroft [69] showed that the acceptance of wind energy depends not only on the perception of low frequency waves and noise, environmental attitude, and experience with wind energy, but also on knowledge. Energy-related knowledge is related to education level. How will higher education levels affect the adoption of renewable energy and nuclear power? Based on the research of 250 randomly selected Hungarian higher education students, Berényi et al. [70] showed that solar energy is appreciated, but confidence in nuclear power is low, except for its future role. These results suggest that general knowledge and education level can perform different functions for different energy sources. The following hypothesis can be made:

**Hypothesis** **9** **(H9).** *The higher the knowledge people have of renewable energy and the lower knowledge they have of nuclear power, the more positive attitude toward policy support and payment intention they will have for the energy transition from nuclear power to renewable energy.*

#### 3.3.4. Trust

Trust is a key influencing factor for energy acceptability. According to Park and Ohm [62], trust has increased the acceptance of renewable energy technology by increasing perceived benefits. Carlisle et al. [55] showed that trust in project developers of solar energy had a positive relationship with support for its development. Park and Ohm [62] showed that perceived trust had indirect positive effects on the acceptance of renewable energy technologies by ways of the perceived benefits and risk. Similarly, according to Whitfield et al. [71], increased trust in nuclear governance institutions reduced the perceived risk of nuclear power and, together with higher trust and lower risk perceptions, predicted positive attitudes toward it. Moreover, Bronfman et al. [58] showed that trust in regulatory institutions retained significant direct and indirect effects on acceptance of energy sources such as fossil, nuclear energy, and hydroelectricity. 

However, trust does not work well under sufficient knowledge of the risk of an event. On the other hand, trust works in particular when there is little knowledge. Therefore, when it is difficult to make accurate decisions based on knowledge, decisions rely on social trust by the appropriate actor [52]. This means that the public uses social trust when making decisions about risks and benefits [48]. Thus, the following hypothesis is proposed:

**Hypothesis** **10** **(H10).** *The higher the trust people have for renewable energy, and the lower the confidence they have for nuclear power, the more positive attitude toward policy support and payment intention they will have for the energy transition from nuclear power to renewable energy.*

## 4. Research Design

### 4.1. Data Collection

The data used in this study were collected in 2019 from 1020 respondents in Korea adopting a multi-stage stratified probability sampling. The questionnaire was designed by the researchers, and the collection of the data was conducted by Hankook Research, a professional survey research company. The name of the survey was “Social Survey on Particulate Matter and Energy”.

Among respondents, 47.6% (*n* = 486) were men, and 52.4% (*n* = 534) were women. By age, 15.8% (*n* = 161), 16.4% (*n* = 167), 20.3% (*n* = 207), and 27.3% (*n* = 278) were in their 20s, 30s, 40s, and 50s. In terms of education level, each 55.7% (*n* = 568) and 44.3% (*n* = 452) were high school graduates and college students and graduates, respectively. Regarding the household income and electricity bill payment, 61.6% (628) of the respondents paid below the mean and 64.6% (659) paid below the mean, respectively.

### 4.2. Measurements

The purpose of this study is to compare and analyze the factors influencing the supportive attitude and intention to pay for the energy transition policy. Therefore, the dependent variable measured the attitude of the respondents to support the energy transition policy and intention to pay the costs for the energy transition at the individual level. To measure the attitude and intention to pay for the transition from nuclear power to renewable energy, the degree of agreement of each of four statements was measured on a 5-point scale (1 = strongly disagree, 2 = slightly disagree, 3 = neutral, 4 = slightly agree, 5 = strongly agree), as shown in Table 1. We refer to the measurement from Li and Hu [72] for attitude and intention.

The independent variables were largely composed of three factors. First, the control variables consisted of demographic variables such as gender, age, and education level (1 = uneducated, 8 = graduate school graduated); social class (1 = lower, 5 = middle, 10 = upper); household income; amount of average monthly electricity bill payment; and perceived energy security (measuring the opinion on three statements using a 5-point scale about disagreement and agreement for each statement). 

Second, the variables in the value factors referred to political ideology (progressive), environmentalism, and S&T optimism. We referred to the measurements of the value variables from Park et al. [21]. We adopted the measurement from Riley et al. [73] for environmentalism and from Kim and Kim [74] for S&T optimism.

The questions were measured on a 5-point scale. As an exception, political ideology was measured on a 10-point scale (“1 = conservative”, “10 = progressive”). Also, for the government support, we asked “How much do you support the Moon Jae-in government?” using a 10-point scale. 

Third, the variables in the perceived factors for renewable energy and nuclear energy included perceived benefits, perceived risks, knowledge, and trust. We adopted the measurements of the perception variables from Wang and Kim [75], Siegrist et al. [76], and Kim et al. [77]. The questions were measured on a 5-point scale (1 = strongly disagree, 2 = slightly disagree, 3 = neutral, 4 = slightly agree, 5 = strongly agree) about each statement.

The reliability of items for each theoretical concept is shown in Table 1.

## 5. Analysis Results and Findings

### 5.1. Basic Analysis

The difference in average value between attitude and intention to pay (3.11 and 2.80, respectively) indicate that the policy support for energy transition issues is higher than the intention to pay because attitude has no costs involved. 

**Figure 2:**
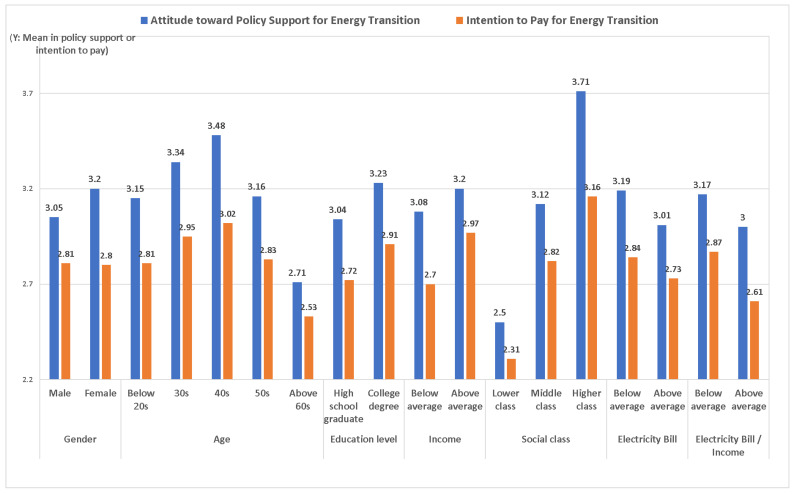
Mean difference in the sociodemographic group.

Next, the mean difference between support attitude and intention to pay according to the control variables are shown in Figure 2. The average value of the measured values was used for support and intention. When evaluating differences between two groups, we used an independent sample *t*-test. When three or more independent groups were analyzed, a one-way ANOVA test was used.

First, women have a higher attitude value toward policy support on energy transition than men (t = −2.288, *p* < 0.01). The t-value measures the size of the difference relative to the variation in data. P is the proven value for statistical significance. In the case of the intention to pay, gender does not show a difference. The reason women have a high support for energy transition policy but low intention to pay may be their poor social and economic status.

In the case of age, people in their 30s and 40s generally have high levels of policy support for energy transition (F = 19.582, *p* < 0.001). The age group shows a difference in intention to pay in the following order: 40s > 30s > 50s > 20s > 60s (F = 9.402, *p* < 0.001). Overall, policy support and intention to pay are high in those in their 30s and 40s, which seems to be because these age groups are more progressive than other age groups, and they are the most economically active, with a considerably high ability to pay.

In the case of education, those who have college or higher education level show more support and intention to pay the energy transition policy than those who have a high school level or those with lower education level (t = −2.881, *p* < 0.01/t = −3.24, *p* < 0.01). A high level of education corresponds to more knowledge of the energy transition and income, hence linking with a higher ability to pay for the energy transition.

In terms of household income, there is no difference between groups of policy support. However, the group with average or higher household income has a higher intention to pay than the group with less than average (t = −4.40, *p* < 0.01). Household income does not affect policy support but makes a difference in the intention to pay. These results prove the existence of the inconsistency that payment intention regardless of attitude can appear in the case of income.

The group in the higher social class shows more attitude toward policy support and higher intention to pay for energy transition than the group in a lower social class (F = 49.981, *p* < 0.001, F = 27.668, *p* < 0.001). This may be because the higher one’s social class, the more resources they have for some things. This is interesting as variables representing the same economic and social status, income, and social class differ in policy support. Although there is no difference in policy support for income, there is a difference in social class. Income reflects more objective material aspects, whereas social class is based on subjective self-evaluation. This difference in policy support seems to reflect a difference in those attributes.

In the case of electricity bill payment, those who pay below-average rates show more policy support than those who pay above average (t = 2.539, *p* < 0.01). The group below the average showed a higher intention to pay than the group above the average (t = 1.72, *p* < 0.05). Regarding the relationship between electricity rates and income, the low payment group shows more policy support (t = 2.184, *p* < 0.01) and higher intention to pay than the high group (t = 3.62, *p* < 0.001). Hence, people with low electricity bills relative to their income are more likely to support policies and pay for an energy transition. Although electricity rates will increase during an energy transition, the results of this study show that those who are relatively less burdened by the actual electricity rate increase support and intend to pay for the energy transition.

### 5.2. Correlation Analysis

To determine the basic relationship between these variables, we executed a Pearson simple correlation analysis between the variables (Table 2). Correlation analysis can measure the relationship between two random variables, and the correlation coefficient measures the strength of the correlation [78]. Pearson correlation coefficients measure only linear relationships [79]. Therefore, they should satisfy the condition for linearity relationships between two variables. On the other hand, Spearman works with rank-ordered variables [79]. The Spearman’s test is therefore useful where basic assumptions of linearity and continuous variables necessary to perform a Pearson’s bivariate correlation analysis have not been met. As we confirmed the linearity of variables, we used Pearson correlation. 

The correlation coefficient between attitude toward policy support and payment intention for energy transition is approximately 0.628. This relatively high coefficient value comes from a scale that commonly contains the energy transition concept. However, since it is not a very high value, there is a possibility of inconsistency between attitude and intention to pay.

When looking at the correlation between attitude toward policy support and other variables, the former had a statistically significant positive relationship with progressivism, Moon Jae-in government support, and environmentalism. Additionally, this attitude showed a positive association with the perceived benefit and trust and a negative relationship with the perceived risk of renewable energy, a positive relationship with the perceived risks, and a negative one with the perceived benefits of nuclear energy.

From the analysis results, first, the variables with the highest correlation values were the perceived benefits of renewable energy. Next, the explanatory power of trust in renewable energy, support for the Moon Jae-in government, and trust in nuclear energy are shown in order. These results showed that energy transition depends on the benefits and trust in renewable energy but also nuclear energy. In energy transition, generally, renewable energy is the final goal for energy transition, and nuclear energy is the target for giving up. In addition, not only perception factors but also political value factors were involved in energy transition. These results suggested that various factors such as benefits, trust, and politics should be considered in the energy transition process. Second, the perceived risks and benefits in nuclear energy and all perception factors in renewable energy played a role in inducing the transition from nuclear to renewable energy. These results suggested that energy transition is possible only when each of nuclear and renewable energy acts as the push and pull factors, respectively. Interestingly, since the coefficient value of perceived risk in nuclear energy is larger than that in renewable energy, it can be inferred that fear of nuclear energy promotes energy transition toward renewable energy. Third, S&T optimism and knowledge variables are not statistically significant. Since these variables presuppose rationality, it suggests the possibility that energy transition will not simply depend on rational judgment. 

Intention to pay for energy transition showed a positive association with progressives and support for the Moon Jae-in government. Environmentalism, which was significant in terms of attitudes, was not significantly related to payment intentions. This implies that although environmentalism contributes to the formation of attitudes, there is a certain limit to its role in inducing action.

When looking at the relationship between intention to pay and renewable energy variables, perceived benefit, knowledge, and trust had a positive relationship with them, but a negative relationship with perceived risk. Also a relatively high correlation was found with trust whereas benefit and low correlation was found with perceived risk and knowledge. Knowledge was not significant toward policy support, but showed a significant impact on payment intention. This suggests that, unlike environmentalism, knowledge may be a driving factor inducing behavior.

In the case of nuclear energy variables, intention to pay shows a positive relationship with perceived risk, knowledge, and trust, but a negative relationship with perceived benefit. In addition, the perceived risk has the largest coefficient values. This shows that the fear of nuclear power exists during the energy transition. Knowledge and trust, which are not significantly different in attitudes, can induce an intention to pay. These results imply that the transition to renewable energy can be achieved not only by the simple energy itself but also in the relationship of competing energies such as nuclear energy.

Related with the intention to pay, the highest coefficient value is trust in renewable energy, followed by support for the Moon Jae-in government, perceived benefits in renewable energy, and perceived risk in nuclear energy. These results suggest that the transition to renewable energy depends not only on renewable energy itself but also on nuclear energy and value factors. 

### 5.3. Regression Analysis

Next, regression analysis was used to examine the determinant structure of the factors affecting the attitude toward policy support and intention to pay for energy transition (Table 3). The following equation represents the main model specified in this study:

$$Y_{i,t}=\alpha +\beta_{1}VF_{i,t}+\beta_{2}PF_{i,t}+\beta_{3}C_{i,t}+\varepsilon$$

where the dependent variable ($Y_{i,t}$) consists of attitude toward policy support for energy transition and intention to pay for energy transition; $\beta_{1}$, $\beta_{2}$, are coefficients denoting the explanatory power of each regressor in the regression model on the dependent variable. $\beta_{1}$ is a vector of the value factor, including political ideology, support for Moon’s government, environmentalism, and S&T optimism. $\beta_{2}$ is a vector of perceived factors, including perceived benefit and risk of renewable energy, perceived benefit and risk of nuclear energy, knowledge and trust of renewable energy, and knowledge and trust of nuclear energy. $\beta_{3}$ is a vector of control variables, including gender, age, education, income, social class (middle), social class (high), electric fee, electric fee/income, and energy security.

To check the linearity of the model, first, we checked the normal probability plot of the regression-standardized residual. We confirmed that there was a normal curve in the plot in the standardized residual. Second, to compare the observed cumulative distribution function (CDF) of the standardized residual to the expected CDF of the normal distribution, we examined normality using a P-P diagram. The P-P diagram in the graph confirms the uniformity and normality of the residuals. As the data were uniformly distributed, the diagram proves the normality of the residuals. Third, we checked the residual plot using a scatter plot. The results showed the distribution is centered at 0 and does not have a specific pattern within a certain range regardless of the value. Fourth, to see the independence of the residuals, the Durbin–Watson statistic was calculated and was found to be 2.075 in Model 1 and 2.009 in Model 2. These figures confirmed that there was independence of residuals. Through the above four checks, it was found that the current model satisfies the linearity or other conditions for regression analysis.

The Model 1 sets support the attitude toward the energy transition as a dependent variable. All of the demographic factors as control variables did not show a significant effect. The fact that the control variables with social structural characteristics are not significant and that the perception and value factors with strong socially constructed attributes are significant suggest that energy transition depends on subjective judgment and shared social discourse.

Among value variables, the more progressive political ideology (B = 0.036, *p* < 0.01), support for the Moon Jae-in government (B = 0.056, *p* < 0.001), and the more environmentalism (B = 0.106, *p* < 0.01), the higher the policy support for energy transition. Among perception variables within renewable energy, the higher the perceived benefit in renewable energy (B = 0.287, *p* < 0.001) and higher trust (B = 0.230, *p* < 0.001), the higher the supporting attitude. Next, in terms of perceptual factors for nuclear power, lower benefit (B = −0.227, *p* < 0.001) and higher perceived risk (B = 0.234, *p* < 0.001) induce higher support for the energy transition. 

When looking at the standardized regression coefficient values, variables that have a significant impact on support attitude toward energy transition could be listed in the order of the perceived benefit of renewable energy (0.254) > perceived benefit of nuclear energy (−0.227) > support for the Moon Jae-in government (0.146) > perceived risk of nuclear energy (0.202) > trust in renewable energy (−0.190) > environmentalism (0.069) > progressive ideology (0.062) > S&T optimism (−0.017). The findings that the explanatory power of perceived benefit is somewhat high suggested the need to specify the cost and gains in the course of the energy transition. The perceived risk in nuclear energy and trust in the government and policies related to renewable energy have a significant impact on energy transition. Three factors—benefit, risk, and trust—play a meaningful role in such a transition. Moreover, when comparing the value and perception factors, all variables within the perception factor have the highest explanatory power. These results imply that the energy transition presents a conflict over realistic interests rather than based on a fundamental philosophy.

Model 2 sets the intention to pay for energy transition as a dependent variable. Demographic or control variables have no significant effects, except social class. Compared to the low class, the middle class (B = 0.133, *p* < 0.01) and high class (B = 0.240, *p* < 0.01) show more active intent to pay for energy transition. 

Out of four value variables, only support for Moon Jae-in showed a significant impact; the more support received by Moon’s government (B = 0.054, *p* < 0.001), the higher the intention to pay for energy transition. The fact that only the support of the regime out of four value variables plays a significant role indicates that the energy transition is a very politicized issue; the transition can be a part of the political game surrounding the political leader. 

In the perceptual factor for renewable energy, the higher perceived benefit (B = 0.103, *p* < 0.01) and trust (B = 0.280, *p* < 0.001) in renewable energy would increase the intention to pay for energy transition. As for the perceptual factor for nuclear power, the lower perceived benefit (B = −0.083, *p* < 0.01) and higher perceived risk (B = 0.148, *p* < 0.01) can increase the intention to pay. For energy transition, structurally, when comparing Model 1 with Model 2, there are four significant variables in perceptual factors. These results imply that homogeneity in influencing factors may exist between attitudes toward policy support and intention to pay.

Based on the standardized regression coefficient values, variables could be listed in the following order: trust in renewable energy (0.252) > support for the Moon Jae-in government (0.154) > perceived risk in nuclear power (0.139) > perceived benefit in nuclear power (0.099) > perceived benefit in renewable energy (−0.087) > social class (high) (0.081) > social class (middle) (0.065). 

The biggest difference between Models 1 and 2 is that in Model 1, the perceived benefit from renewable energy as the highest explanatory power, and in Model 2, trust in renewable energy has the highest explanatory power. Another difference is that many value variables were significant in Model 1, but their influence disappeared except for support for the government in Model 2. This suggests that value variables have limitations in inducing behavior. The common variables in Models 1 and 2 include support for the Moon Jae-in government and perceived benefits and risks of nuclear energy. These results suggest that to advance the energy transition, the emphasis should differ between trust, risk, and benefits. 

### 5.4. Logistic Regression

We executed binary logistic regression analysis to examine the impact of value and perception factors on the probability of belonging to one of four groups, based on inconsistency between attitude and intention. For this purpose, we divided the respondents into two groups according to support attitude and payment intention by using the mean value. Then, we created four groups by mixing the high and low groups in each mean value in attitude and intention. The four groups consisted of low attitude and intention to pay comprising 296 (29.0%) out of 1020 people and high attitude and intention comprising 376 (36.9%). On the other hand, the group with the lowest support and high intention to pay was 254 (24.9%). The group with high support and low intention was 94 (9.2%). To find the probability of belonging to each group, we made the dummy variable in which three groups were set as the reference group with a value of 0 and one group as the target group with a value of 1.

When the outcome of interest was a binary variable, logistic regression was appropriate [80]. The next equation represents the logistic regression model specified in this study:

$$Y_{i,t}=\alpha +\beta_{1}VF_{i,t}+\beta_{2}PF_{i,t}+\beta_{3}C_{i,t}+\varepsilon$$

where $Y_{i,t}$ denotes a dependent variable; $\beta_{1}$ is a vector of value factors, including Political ideology, support for Moon’s government, environmentalism, and S&T optimism; $\beta_{2}$ is a vector of perceived factors, including perceived benefit and risk of renewable energy, perceived benefit and risk of nuclear energy, knowledge and trust of renewable energy, and knowledge and trust of nuclear energy. $\beta_{3}$ is a vector of control variables, including gender, age, education, income, social class (middle), social class (high), electric fee, electric fee/income, and energy security. The analysis results appear in Table 4.

In the case of Model 3, the group with low support and intention to pay is the focus group, whereas the remaining three groups are the reference groups for comparison. This group seems to logically think and behave because there is consistency between attitude and intention. Those who have high scientific optimism and perceived benefits in nuclear energy are more likely to belong to this group, whereas if respondents are in the middle class or politically progressive and have a higher risk perception of nuclear energy or perceived benefits and trust in renewable energy, then they are less likely to belong to this consistent group. This finding suggests that if there is a higher perceived benefit and lower perceived risk, people tend to refuse to pay the costs for the energy transition. On the other hand, high perceived benefits and trust in renewable energy are likely to induce support and intention to pay for energy transition. This finding indicates that perception of each of nuclear and renewable energy plays opposite roles in determining the attitude and intention toward energy transition. Those who have progressive ideology show a higher probability of belonging to a group with low support and intention group. This suggests that the ideological role that emerged in the Western energy transition process also exists in Korea, suggesting that ideological conflicts will occur during the energy transition process.

The group in Model 6 is contrary to the one in Model 3. As shown in Model 3, current government support, social class, perceived benefits and trust in renewable energy, and perceived benefit and risk in nuclear power show a significant impact on the probability of affiliation with this group. Variables that were not significant in Model 3 appear significant—educational background, political ideology, and environmentalism. Conversely, S&T optimism lost its statistical significance in Model 6. This difference suggests that the impact of independent variables on the possibility of belonging to two consistent groups may not be symmetric. This fact can also be confirmed through the order of magnitude of the exponent value; in Model 3, perceived benefits in nuclear power > S&T optimism > political ideology, whereas in Model 6, social class > perceived risk in nuclear power > perceived benefit in renewable energy > trust in renewable energy. These results suggest that the influence of predictors on the probability of belonging to two consistent groups in Model 3 and Model 6 is asymmetrical.

Models 4 and 5 show inconsistency in attitude and behavioral intention. In particular, Model 4 is logically unusual in that it has low support for energy transition policies but expresses a high intention to pay. This is an example of the coexistence of negative attitude and positive intention. Among the predictors, if respondents show high environmentalism and perceive benefits of renewable energy, they have a high probability of belonging to this group. However, the more that respondents are politically progressive, the more knowledge they have of renewable energy, and the more they perceive benefits of nuclear power, the lower the probability of belonging to this group. Environmentalism is a strong driver of inducing action even without policy support. However, knowledge acts as a factor that suppresses the inconsistent choice between low support and high intention to pay. With the same perceived benefit, renewable energy acts as a pull factor that increases the possibility of belonging to this group, while perceiving benefits of nuclear power acts as a push factor that lowers it.

Model 5 is about a group with high support for the policy but low intention to pay for the energy transition. If the risk perception of renewable energy is high, the probability of belonging to this group is high. Since those with older age are related to a higher perceived risk in nuclear energy and environmentalism, they have a lower probability of belonging to this group. Even if the support is high, the reason that the intention to pay is low may come from a lack of resources to pay or a lack of a strong will to maintain an attitude to behavior. It is inferred that an educational background will provide such a resource.

When comparing the four models, first, it can be noted that there is no model with the same determinant structure. The fact that the size and significance of the factors affecting the affiliation of the four groups in four models differ suggests that different approach strategies are needed for managing each group for inducing energy transition. Second, when there are significant variables in Model 3 to Model 6, there is a difference in the range of significance for each independent variable. First, no variables influence the four models together. Environmentalism, perceived risk of renewable energy, and perceived benefit and risk of nuclear power have a significant effect in the three models, and age, political ideology, S&T optimism, and knowledge of renewable energy have a significant effect in only one model. These results suggest that independent variables have universality and particularity in the explanatory power of the affiliation of the group. Third, among the independent variables, there are cases where the influence of independent variables is asymmetric. For example, the educational background has an effect on an attitude of high support but not on an attitude of low support. Fourth, from the perspective of consistency between attitude and behavior, Models 3 and 6 are consistent, whereas Models 4 and 5 are inconsistent. Among the independent variables, social class (middle), current government support, and trust in renewable energy affect only Models 3 and 6, whereas age and the perceived risk in renewable energy affect only Models 4 and 5. The former variables play a role in inducing the consistency of attitude–action intention, while the latter one induces inconsistency of it.

In short, the above results show that there may be inconsistency between attitude and behavior, and the scope and degree of influence of independent variables affecting them are different.

## 6. Discussion and Implications

Our analysis provides several implications in terms of theory and practice. In terms of theory, looking at the flow of existing studies on the causes of differences between attitudes and behavioral intentions, the causes were often explained by the theory of planned behavior or reasoned behavior [81,82,83]. Recently, new approaches or theories have developed to explain the inconsistency between attitudes and behaviors. For example, based on the psychometric paradigm, perceived risk has been demonstrated to function as a major variable explaining the gap between attitude and behavioral intention [84]. VBN theory has also been used to explain the gap between attitude and intention to pay [85]. VBN theory differs from TPB theory in that TPB theory considers rational choice while VBN emphasizes values and moral norms [86]. The VBN theory is related to the Norm Activation Theory (NAM), which is a theory that pro-environmental behavior is achieved by activating personal norms [87]. Behavioral Reasoning Theory (BRT) [88] is also one of the theories used in explaining the relationship between attitude and behavior [89].When existing theories explaining the process of attitude leading to behavior, above theories, they mainly focused on norm or value. Some theorists argued that it needs a holistic approach [90,91,92]. Several studies have recently emphasized other variables except value or norm. For example, information and mechanisms [93] and emotions [94] are emphasized as variables that account for the differences between attitudes and behaviors. Additionally, social identity can function as a factor that explains the difference between attitude and behavior [95]. Also, some studies reexamined the relations between attitude-intention-behaviors. For example, Kim and Hunter [96] showed that (a) the A–BI (attitude–behavioral intentions) correlation was higher than the A–B (attitude–behavior) correlation, (b) the BI–B (behavior) correlation was higher than the A–B correlation, (c) the A–BI correlation was higher than the BI–B correlation, (d) the variation in BI–B correlations was greater than that of A–BI, and (e) attitudinal relevance influenced the magnitude of the A–BI correlation.

Previous studies have been insufficient to integrally consider the value factors covered in VBN theory or the perception-dimensional factors emphasized in TPB theory, and psychometric paradigms. Some studies focus only on norm or control in TPB theory, on value VBN theory, or on perception in the psychometric paradigm. So value and perception are not considered fully. In addition, as each study focuses partially on individual variables such as emotional aspects, social identity, and information, so it can be said that it has limits to considering the overall influence of them at once. Therefore, this study simultaneously reflected the value element and the perception element in the model construction.

In addition, existing studies show limitations because they do not perform group-specific comparisons that can compare differences based on the matrix between attitudes and behavioral intentions. This study attempted to supplement the limitations through logit regression analysis that can compare such differences.

The theoretical contributions of this study can be evaluated by comparing with existing studies. First, recent previous studies have shown that inconsistency occurs in real situations, not ethical ones. For example, Lin and Shi [97] demonstrated that the inconsistency between the intention to purchase new energy vehicles and behavior is more obvious than in other ethical contexts. Our study demonstrated the existence gap between attitude and intention in the field of energy transition.

Second, there have been a lot of studies on the relationships between attitude-intention and gaps with respect to norms [98]. Parker et al. [99] showed that moral norms enhanced the prediction of intentions to perform various driving behaviors over and above attitudes and perceived behavioral control. Additionally, participants whose intentions were more aligned with their moral norm tended to perform the behaviors compared with participants whose intentions were more related to their attitude. Vermeir and Verbeke [100] showed that despite rather negative personal attitudes, experiencing social pressure from peers (social norm) explains intentions to buy. Values are similar to norms, but research on them has been lacking. However, values are fundamental orientations that can influence attitudes and intentions on par with or beyond norms. For example, in terms of ideology, Marquart-Pyatt et al. [101] showed that not only energy attitudes but also political ideology shape energy policy preferences and behavioral intention. Kim et al. [102] confirmed the role of conservative ideology in judging the energy preference. Our studies confirmed not only the role of progressive political ideology but also the role of government support and S&T optimism. On the other hand, according to Mishal et al. [103], results indicate that environmental consciousness has an influence on green purchasing attitude and perceived customer effectiveness; finally links to green behavior have an influence on green purchasing behavior. Moreover, Wang et al. [83] demonstrated that the attitude–intention gap is explained by behavioral reasoning theory. Additionally, environmental values affect both reasons and green consumption attitudes. However, those studies showed only that environmentalism had an effect as an indirect variable. This study revealed that environmentalism affects behavior as a direct variable. 

Third, there are a number of studies that emphasize a sense of control or efficacy in existing studies. For example, Lin and Shi [104] showed that consumers’ perceived reduction in policy effectiveness and perceived behavior control can increase and decrease the intention behavior gap, respectively. Vermeir and Verbeke [99] showed that involvement with sustainability, certainty, and PCE (perceived consumer effectiveness) has a significant positive impact on attitude towards buying sustainable dairy products, which in turn correlates strongly with the intention to buy. However, although a more direct utility variable than efficacy may be perceived cost, risk, and benefit, few studies have investigated the role of these variables. There have been a few recent studies that have focused on perceived benefits or risks. Mishal et al. [101] showed that the gap in the translation of eco-conscientiousness into green behavior and green purchase behavior can be attributed to costliness, non-availability with less variety, lack of brand reputation of green products, and budget constraints for customers. According to He et al. [104], high policy awareness and high risk preferences can narrow the intention–behavior gap in bioenergy production. In this study, it was possible to confirm the significant role of perceived benefits and risks in explaining the behaviors in energy transition.

Finally, recently, trust has been used to explain something as a repertoire variable that appears all the time. For example, Campbell and Fairhurst [105] showed that trust was found to moderate the relationship between purchase intentions and the extent of purchase for locally produced foods. Our study shows that trust directly affects attitude and intention coherence.

Our finding gives several practical implications. First, value and perception factors simultaneously affect the attitude and intention to pay for the energy transition. Moreover, structural social class in sociodemographic factors only affects payment intention, not attitude. The intention to pay is possible only when there is a middle or upper social class. To induce energy transition, we should consider three factors, i.e., perception, attitude, and social structure, in a balanced manner.

Second, at the variable level, ideology and S&T optimism, as value factors, and perceived benefits, perceived risks, and trust, as perceptual factors, simultaneously influence the attitude toward policy support and payment intention for the energy transition. Significant variables affecting attitude and behavioral intention have something in common. Several perception variables in both renewable and nuclear energy have an effect on the supportive attitude and payment intention for the energy transition. This finding implies that the energy transition toward renewable energy is not a simple problem of renewable energy itself but is closely related to nuclear energy. These results suggest that the relationship between energy sources should be considered for energy policy change.

Third, when considering the significance of perception variables in renewable and nuclear energy, the perceived benefits and trust in renewable energy have a great impact on two dependent variables. Each trust in renewable energy and perceived risks in nuclear energy differentially affect attitude and intention. This suggests that trust is a pull factor for inducing the energy transition to renewable energy, whereas perceived risk acts as a push factor for distancing from nuclear energy.

Fourth, when looking at the explanatory power of independent variables, the attitude toward policy support for energy transition was mainly influenced by the perceived benefits in nuclear power > perceived benefits in renewable energy > perceived risks in nuclear power; in the case of the intention, trust in renewable energy > the perceived risk in nuclear power > perceived risk in renewable energy. These results provide evidence that the structure of the influence between attitude and intention may be different. This suggests that priorities on the policy should be different when approaching the attitude and intention to pay for the energy transition.

Finally, through logistic analysis, there are groups that show not only consistency but inconsistency between supportive attitudes toward the energy transition and the intention to pay for it. Among the inconsistent groups, groups support energy transition policies but do not have the intention to pay for the transition costs. Moreover, the variables from value and perception have a structural effect on such an inconsistent response. To promote the energy transition, it is necessary to actively respond to these groups, in particular, those who express a supportive attitude toward the transition but do not pay the cost.

## 7. Conclusions

This study aimed to compare and analyze the factors influencing the attitude of the public toward policy support and payment intention with respect to the transition from nuclear power to renewable energy. This study set hypotheses that a supportive attitude may not necessarily lead to behavioral intention. To this end, a supportive attitude toward the energy transition policy and the intention to pay for it were set as dependent variables. The determinant structure and explanatory power of the independent variables (e.g., sociodemographic, value, renewable energy perception, and nuclear power perception factors) were analyzed. In addition, to analyze the inconsistency between attitude and intention, respondents were classified into four groups for logistic analysis. 

This study was able to (1) identify the structural variables that affect the attitude and intention for energy transition, (2) discern the existence of consistency and inconsistency between attitude and intention, and (3) determine which variables influence those (in)consistencies. The results of these studies can have theoretical and practical implications for the promotion of energy transition policies in the future. Our findings predict that there will be some social conflicts around energy transition because of the inconsistency of human behavior. Karimian et al. [106] argued that sustainability aims to balance economic growth, environmental issues, and the social and welfare condition of a city to meet the present needs without jeopardizing the resources and future generations’ opportunities.

It is worth noting the several limitations of this study. Since the focus is on payment intention rather than payment behavior, there is uncertainty about whether the results will be implemented in actual actions in a real context. Second, since this study focuses on the energy transition from nuclear power to renewable energy, it does not refer to various transition paths. Third, in the case of logistic analysis, setting a dummy variable as an independent variable can be configured more variously than the current one. Fourth, our studies did not fully consider communication factors [107,108,109,110,111], economic or resource factors [111,112], or structural factors [54,113,114]. Finally, further work needed for highlighting the causal model [115,116].

## Figures and Tables

**Table 1:** Sample characteristics.

	Variable		Statement	Scale	Cronbach’s a
Dependent variable	Attitude toward policy support for energy transition		Abandoning nuclear power and moving to renewable energy is the way to go.	5-point scale (1 = Strongly disagree, 5 = Strongly agree)	0.866
			The pace of transition from nuclear power to renewable energy should be accelerated.		
	Intention to pay for energy transition		If the transition from nuclear energy to renewable energy involves costs, I personally have the intention to pay them.		0.855
			If I must pay more for electricity to reduce nuclear energy, I have the intention to pay more.		
Value factor	Political Ideology (progressive)		When we divide political ideology into progressives and conservatives, the most conservative is 1 point, and the most progressive is 10 points. How much do you think it is?	10-point scale (1 = conservative, 10 = progressive)	-
	The Moon Jae-in government support		To what extent do you support the Moon Jae In government?	10-point scale (1 = not support at all, 10 = strongly support)	-
	Environmentalism		Currently, the earth is facing a serious environmental and ecological crisis.	5-point scale (1 = Strongly disagree, 5 = Strongly agree)	0.808
			The earth has already exceeded its own limits.		
			Nature is so sensitive that it is easily destroyed.		
	S&T optimism		Technology makes our lives healthier and more convenient than causing problems.		0.671
			Thanks to advances in science and technology, the resources present on Earth will be abundant rather than depleted.		
Perceive factor	Renewable energy	Perceived benefit	Renewable energy can be supplied cheaply and stably.		0.827
			Renewable energy contributes to the development of the national economy.		
		Perceived risk	Renewable energy is more dangerous than you think.		0.856
			Renewable energy is very likely to be an accident.		
		Knowledge	I can explain to others the issues related to renewable energy well.		0.899
			I am well aware of policies and issues related to renewable energy.		
		Trust	I trust the government department in charge of renewable energy.		0.888
			I trust the government’s renewable energy policy.		
	Nuclear energy	Perceived benefit	Nuclear energy can be supplied cheaply and reliably.		0.858
			Nuclear power contributes to the nation’s economic development.		
		Perceived risk	The Fukushima nuclear accident is a very serious problem that cannot be compared to any other accident.		0.908
			The Fukushima nuclear accident is a very serious problem considering the situation in Korea.		
			I could be damaged by the Fukushima nuclear accident.		
			I am worried that my family will be damaged by radiation from the Fukushima nuclear power plant.		
			The Fukushima nuclear accident will cause a catastrophe for mankind.		
		Knowledge	I can explain to others the issues related to nuclear power well.		0.873
			I am aware of policies and issues related to nuclear power.		
		Trust	I trust the government department in charge of nuclear power		0.775
			I trust the government’s nuclear policy.		

**Table 2:** Pearson simple correlation.

			1	2	3	4	5	6	7	8	9	10	11	12	13	14
Attitude toward policy support		1	1													
Intention to pay		2	0.628 ***	1												
Value factor		3	0.342 ***	0.287 ***	1											
		4	0.474 ***	0.408 ***	0.597 ***	1										
		5	0.164 ***	0.038	−0.011	−0.011	1									
		6	−0.044	0.033	−0.021	−0.020	−0.059	1								
Perception factor	Renewable energy	7	0.538 ***	0.362 ***	0.177 ***	0.297 ***	0.195 ***	0.071 **	1							
		8	−0.224 ***	−0.119 ***	−0.120 ***	−0.185 ***	−0.043	0.080 **	−0.224 ***	1						
		9	0.028	0.172 ***	0.019	0.018	−0.046	0.175 ***	0.078 **	0.159 ***	1					
		10	0.481 ***	0.466 ***	0.234 ***	0.400 ***	0.037	0.068 **	0.531 ***	−0.180 ***	0.252 ***	1				
	Nuclear energy	11	−0.399 ***	−0.222 ***	−0.210 ***	−0.303 ***	0.094 **	0.154 ***	−0.177 ***	0.069 **	0.018	−0.170 ***	1			
		12	0.459 ***	0.296 ***	0.204 ***	0.296 ***	0.307 ***	−0.044	0.335 ***	−0.165 ***	−0.098 **	0.224 ***	−0.167 ***	1		
		13	−0.043	0.118 ***	−0.011	−0.006	−0.034	0.160 ***	−0.015	0.100 ***	0.630 ***	0.097 **	0.142 ***	0.012	1	
		14	0.052	0.184 ***	0.032	0.161 ***	−0.062 **	0.180 ***	0.133 ***	0.024	0.129 ***	0.399 ***	0.191 ***	−0.034	0.120 ***	1

* *p* < 0.05, ** *p* < 0.01, *** *p* < 0.001. (1) Attitude toward policy support for energy transition, (2) Behavioral intention to pay for energy transition, (3) Policy ideology (progressive), (4) present Moon Jae-in government support, (5) Environmentalism, (6) S&T optimism, (7) Perceived benefit of renewable energy, (8) Perceived risk of renewable energy, (9) Knowledge of renewable energy, (10) Trust for renewable energy (11) Perceived benefit of nuclear energy, (12) Perceived risk of nuclear energy, (13) Knowledge for nuclear energy, (14) Trust for nuclear energy.

**Table 3:** Regression analysis.

			Model 1: Attitude toward Policy Support for Energy Transition			Model 2: Intention to Pay for Energy Transition		
			B	SE	β	B	SE	β
(instants)			0.612	0.262	-	0.345	0.285	-
Control factor		Gender	0.015	0.051	0.007	0.024	0.055	0.012
		Age	−0.002	0.002	−0.028	−0.002	0.002	−0.033
		Education	0.053	0.051	0.025	0.067	0.055	0.034
		Income	0.000	0.000	0.023	0.000	0.000	0.049
		Social class (middle)	0.042	0.058	0.019	0.133 **	0.064	0.065
		Social class (high)	0.082	0.089	0.025	0.240 **	0.097	0.081
		Electric fee	−0.000	0.000	−0.006	−0.000	0.000	−0.039
		Electric fee/income	0.005	0.368	0.000	0.060	0.401	0.004
		Energy security	0.060	0.037	0.036	0.001	0.040	0.001
Value factor		Political ideology (progressive)	0.036 **	0.016	0.062	0.026	0.017	0.048
		Moon’s gov. support	0.056 ***	0.012	0.146	0.054 ***	0.013	0.154
		Environmentalism	0.106 **	0.037	0.069	−0.003	0.040	−0.002
		S&T optimism	−0.025	0.033	−0.017	0.005	0.036	0.004
Perceived factor	Renewable energy	Perceived benefit	0.287 ***	0.031	0.254	0.103 **	0.034	0.099
		Perceived risk	−0.057	0.029	−0.046	−0.006	0.032	−0.005
		Knowledge	−0.003	0.037	−0.003	0.063	0.040	0.058
		Trust	0.230 ***	0.037	0.190	0.280 ***	0.040	0.252
	Nuclear energy	Perceived benefit	−0.227 ***	0.027	−0.219	−0.083 **	0.029	−0.087
		Perceived risk	0.234 ***	0.030	0.202	0.148 ***	0.032	0.139
		Knowledge	−0.017	0.035	−0.014	0.070	0.039	0.063
		Trust	−0.026	0.031	−0.022	0.062	0.034	0.056
N			1020			1020		
R^2^			0.530			0.339		
adj. R^2^			0.520			0.325		
F(*p*)			56.568 (0.000 ***)			24.360 (0.000 ***)		

Note: B = standardized regression coefficient, SE = standard error of the regression, β = β standardized regression coefficient. * *p* < 0.05, ** *p* < 0.01, *** *p* < 0.001.

**Table 4:** Logistic regression.

			Group with Low Supportive Attitude toward Energy Transition						Group with High Supportive Attitude toward Energy Transition					
			Model 3: Group with Low Intention			Model 4: Group with High Intention			Model 5: Group with Low Intention			Model 6: Group with High Intention		
			B	SE	exp(β)	B	SE	exp(β)	B	SE	exp(β)	B	SE	exp(β)
(instants)			4.008	0.998	55.032	−1.857	1.511	0.156	1.810	0.868	6.110	−9.538	1.074	0.000
Control factor		Gender	0.004	0.177	1.004	−0.040	0.272	0.961	0.055	0.162	1.057	−0.143	0.173	0.867
		Age	0.008	0.006	1.008	−0.006	0.009	0.994	−0.016 **	0.006	0.984	0.003	0.006	1.003
		Education	−0.040	0.178	0.961	0.110	0.269	1.116	0.317*	0.163	1.373	−0.368 **	0.170	0.692
		Income	0.000	0.000	1.000	0.000	0.000	1.000	0.000	0.000	1.000	0.000	0.000	1.000
		Social class (middle)	−0.747 **	0.328	0.474	0.224	0.491	1.251	−0.141	0.300	0.868	0.679 **	0.305	1.972
		Social class (high)	−0.317	0.289	0.729	0.068	0.426	1.070	−0.409	0.254	0.664	0.443	0.251	1.557
		Electric fee	0.000	0.000	1.000	0.000	0.000	1.000	0.000	0.000	1.000	0.000	0.000	1.000
		Electric fee/income	−0.783	1.055	0.457	−1.068	4.050	0.344	0.850	1.044	2.339	−4.429	5.838	0.012
		Energy security	−0.107	0.132	0.899	0.166	0.186	1.180	−0.160	0.124	0.852	0.187	0.134	1.206
Value factor		Progressive ideology	−0.033	0.058	0.968	−0.021	0.083	0.980	−0.054	0.052	0.948	0.133 **	0.059	1.142
		The Moon’s gov. support	−0.120 **	0.040	0.887	−0.071	0.061	0.931	−0.021	0.037	0.979	0.159 ***	0.041	1.172
		Environmentalism	−0.088	0.131	0.916	0.139	0.194	1.149	−0.387 **	0.118	0.679	0.342 **	0.132	1.407
		S&T optimism	0.044	0.121	1.045	0.189	0.160	1.208	−0.099	0.111	0.906	−0.059	0.114	0.943
Perceived factor	Renewable energy	Perceived benefit	−0.456 ***	0.109	0.634	0.492 **	0.171	1.636	−0.037	0.103	0.963	0.428 ***	0.113	1.534
		Perceived risk	0.027	0.111	1.027	−0.219	0.164	0.804	0.235 **	0.095	1.265	−0.099	0.105	0.906
		Knowledge	−0.069	0.132	0.934	−0.400 **	0.187	0.670	0.097	0.120	1.102	0.146	0.126	1.157
		Trust	−0.587 ***	0.136	0.556	−0.252	0.181	0.777	0.229	0.124	1.258	0.415 **	0.131	1.514
	Nuclear energy	Perceived benefit	0.550 ***	0.106	1.733	−0.388 **	0.133	0.679	−0.010	0.089	0.990	−0.241 **	0.096	0.786
		Perceived risk	−0.392 ***	0.103	0.676	0.260	0.163	1.296	−0.292 **	0.094	0.747	0.676 **	0.111	1.965
		Knowledge	−0.179	0.132	0.836	−0.186	0.176	0.830	−0.014	0.116	0.986	−0.020	0.124	0.980
		Trust	0.019	0.117	1.019	−0.075	0.167	0.927	0.082	0.102	1.085	0.138	0.114	1.148
N			1020			1020			1020			1020		
Accuracy			77.9%			92.1%			74.4%			76.5%		
*x* **^2^**			5.134 ***			62.988 ***			80.545 ***			350.153 ***		
−2LL			941.214			504.653			1098.415			974.314		
Cox & Snell			0.261			0.063			0.076			0.291		
Nagelkerke R^2^			0.369			0.146			0.111			0.400		

* *p* < 0.05, ** *p* < 0.01, *** *p* < 0.001.

## Data Availability

The data presented in this study are available on request from the corresponding author. The data are not publicly available due to regulations and guidelines regarding the data.
